# Microbiota Plays a Role in Oral Immune Priming in *Tribolium castaneum*

**DOI:** 10.3389/fmicb.2015.01383

**Published:** 2016-01-06

**Authors:** Momir Futo, Sophie A. O. Armitage, Joachim Kurtz

**Affiliations:** Institute for Evolution and Biodiversity, University of MünsterMünster, Germany

**Keywords:** microbiota, oral infection, immune priming, innate immunity, *Tribolium castaneum*, *Bacillus thuringiensis*

## Abstract

Animals are inhabited by a diverse community of microorganisms. The relevance of such microbiota is increasingly being recognized across a broad spectrum of species, ranging from sponges to primates, revealing various beneficial roles that microbes can play. The red flour beetle *Tribolium castaneum* represents a well-established experimental model organism for studying questions in ecology and evolution, however, the relevance of its microbial community is still largely unknown. *T. castaneum* larvae orally exposed to bacterial components of the entomopathogen *Bacillus thuringiensis* bv. *tenebrionis* showed increased survival upon a subsequent challenge with spores of this bacterium. To investigate whether *T. castaneum* microbiota plays a role in this phenomenon, we established a protocol for raising microbe-free larvae and subsequently tested whether they differ in their ability to mount such a priming response. Here we demonstrate that larvae with significantly lowered microbial loads, show decreased survival upon secondary challenge with *B. thuringiensis* bv. *tenebrionis* spores, compared to animals that were allowed to regain their microbiota before priming. Although the exact mechanism of oral immune priming is unclear, we here suggest that microbiota plays a crucial role in oral immune priming in this species.

## Introduction

Parasites and infectious diseases impose a serious threat to the fitness of their hosts. To defend themselves, vertebrates have evolved an innate and adaptive immune system, the latter being characterized by specificity and memory (Janeway et al., 2004). The degree to which invertebrates also possess a form of immune memory has been an intensively debated topic over the last decade (Kurtz, 2004; Little et al., 2005; Rowley and Powell, 2007). Contrary to earlier dogma that invertebrates are incapable of adaptive immune responses, evidence has accumulated that many arthropod species show a phenomenon named “immune priming” (Schmid-Hempel, 2005), i.e., enhanced protection following a prior exposure to a pathogen, which is today recognized as a form of immune memory (Kurtz and Franz, 2003).

Although a phenomenon not generally characteristic for all invertebrate-parasite systems that have been studied, immune priming has been repeatedly shown in a number of arthropod species, especially insects. Evidence has been found for priming using diverse infection routes by which parasites can enter the body of various insect species, e.g., direct hemocoelic priming and subsequent cuticular infection in *Tenebrio molitor* (Moret and Siva-Jothy, 2003) or oral priming in our model organism, *Tribolium castaneum* (Milutinović et al., 2014). Furthermore, evidence for a range of specificities in immune priming, including highly specific responses, has been found when the infection was done by direct introduction into the hemocoel of *Bombus terrestris* (Sadd and Schmid-Hempel, 2006), *Drosophila melanogaster* (Pham et al., 2007) and *T. castaneum* (Roth et al., 2009), or via oral infection of a copepod host *Macrocyclops albidus* (Kurtz and Franz, 2003). It is interesting to note that in the same experimental setup, hosts can show a primed response against some pathogens, but not against others (Pham et al., 2007; Roth et al., 2009), while in other systems the whole phenomenon can be absent (Reber and Chapuisat, 2012). In a few studies, immune priming has also been shown to act across generations (Little et al., 2003; Sadd et al., 2005; Roth et al., 2010; Eggert et al., 2014, 2015).

The mechanistic underpinnings of immune priming in arthropods are, in general, poorly understood. However, after direct hemocoelic priming phagocytosis was shown to be involved in *D. melanogaster* (Pham et al., 2007) and the woodlouse, *Porcellio scaber* (Roth and Kurtz, 2009), furthermore activation of the Toll signaling pathway was implicated for *D. melanogaster* (Pham et al., 2007). In the lepidopteran *Galleria mellonella* and the honey bee *Apis mellifera*, another mechanism seems responsible for maternal trans-generational immune priming: the egg-yolk protein vitellogenin binds bacterial cell wall components and carries them to the eggs (Freitak et al., 2014; Salmela et al., 2015).

It has been proposed that the gene *Dscam1*, which produces extreme phenotypic diversity (Schmucker et al., 2000) could play a role in immune priming, potentially providing an explanation for some aspects of specificity, however, this hypothesis has not been directly tested (Armitage et al., 2015). The mechanisms underlying priming via oral infections might be expected to differ, at least in part, compared to hemocoelic infection. This is because the parasites must traverse the gut wall and face the various host defenses (Lemaitre and Hoffmann, 2007) before they can gain entry into the hemocoel. However, an additional level of complexity arises when infections are done orally because prior to passing the gut epithelium, parasites will be subjected to the complex community of bacteria that are known to be harbored in the gut (Douglas, 2015).

It was recently shown that *Tribolium castaneum* (Herbst 1797) larvae show increased survival after oral exposure to entomopathogenic bacterial spores of *Bacillus thuringiensis* subsp. *morrisoni* bv. *tenebrionis* when previously orally primed with the supernatant taken from a spore culture of the same bacterium (Milutinović et al., 2014). *Bacillus thuringiensis* (Berliner 1915) (*Bt*) is a Gram-positive, endospore forming bacterium expressing plasmid-encoded crystalline inclusions (Cry toxins) specifically toxic to various insect orders after ingestion (reviewed, in Palma et al., 2014). For example, the strain used by Milutinović et al. (2014) shows specificity to Coleopteran insects (Oppert et al., 2011; van Frankenhuyzen, 2013). The mechanisms underlying this example of immune priming are unknown. Of particular interest is the question of whether the gut microbiota might influence the priming effect.

The rapid development of modern, culture-independent, molecular methods of microbial identification such as various deep sequencing techniques, have contributed immensely to our understanding of the composition and functional diversity of different microbial communities, i.e., microbiota, associated with a wide spectrum of vertebrates and invertebrates, especially insects (Shi et al., 2010). Indigenous microbes are today considered to be inseparably associated with all living organisms (McFall-Ngai et al., 2013), governing or aiding many biological, ecological and evolutionary processes (Rosenberg and Zilber-Rosenberg, 2013). The importance of insect-associated microbes mostly lies in the gut-harbored bacterial communities (reviewed, in Dillon and Dillon, 2004; Engel and Moran, 2013; Douglas, 2015). Apart from having diverse roles in digestion, communication, reproduction, etc., commensal gut microbes have been shown responsible for shaping the immunological responses of insect hosts in various ways.

In this context, it is of particular relevance to our study that eradication of gut microbiota in adult *Anopheles gambiae* mosquitos eliminated immune priming for resistance against protozoan *Plasmodium falciparum* parasites, which are ingested during blood feeding (Rodrigues et al., 2010). A recent study showed that lipoxin/lipocalin induced hemocyte differentiation triggered by ookinete midgut invasion of *A. gambiae* is responsible for the priming response (Ramirez et al., 2015). However, priming was recently shown to be functional also in the absence of gut microbiota in a different mosquito-malaria system, *Anopheles albimanus* infected with *Plasmodium berghei* (Contreras-Garduño et al., 2015).

It is thus currently unclear whether the involvement of gut microbiota in oral immune priming is specific to the particular system of *A. gambiae* and *P. falciparum*, or can be seen as a more general mechanism of immune priming also in other insect-parasite systems. As a mosquito-protozoan interaction clearly differs in many aspects from the beetle-bacteria host-parasite system, our experiments were designed with the aim of determining whether the indigenous larval microbiota plays a role in oral immune priming in the red flour beetle. Because microbiotas have been suggested to shape the immunological responses of their hosts, we hypothesized that the eradication of the commensal bacteria might lead to the loss of the priming effect.

## Materials and Methods

### Model Organisms

In this study we used the *T. castaneum* Cro 1 population that was established from 165 beetle pairs wild-collected in Croatia in 2010 (Milutinović et al., 2013). Individuals were maintained in overlapping generations at a population size of approximately 3,000 individuals. As a food source and substrate we used organic wheat flour (Alnatura, type 550) with 5% brewer’s yeast (hereafter called standard flour), which prior to use was frozen (-20°C) and subsequently heated (75°C). The population was kept under controlled environmental conditions: 30°C, 70% humidity and on a 12-h light-dark cycle (in the following denoted as standard breeding conditions). For infections we used the bacterium strain *Bacillus thuringiensis* subsp. *morrisoni* bv. *tenebrionis* (*Btt*) (Bacillus Genetic Stock Center, BGSC, Ohio State University, Columbus, OH, USA), stored in 25% glycerol stocks at -80°C before the experiments started.

### Experimental Design

To test for the effect of microbiota on oral immune priming, for every individual we always first removed the natural microbiota of *T. castaneum* by bleaching eggs and raising larvae under sterile conditions. We then allowed the larvae to either be colonized with microbiota or not, by using three types of flour: “untreated,” “sterilized,” or “recolonized” (see below for details). The “untreated” and “recolonized” flour were expected to contain microbes capable of larval gut colonization. After that, we performed priming-challenge experiments with these differently treated larvae. We performed two independent experiments, the methods of which were identical unless described otherwise, however, in Experiment 2 we used eight independently replicated flour treatments for each of the three different flour types.

### Production of Larvae Under Sterile Conditions

For each experiment, a subpopulation of ca. 2,000 approximately 1-month-old adult beetles was allowed to copulate and oviposit for 24 h. The beetles were kept under standard breeding conditions in approximately 500 g of standard flour in a 4.0 L transparent plastic box (Curver, New Grand Chef) with six holes (3 cm in diameter) punctured into the lid and plugged with foam stoppers (4.2 cm in diameter) (K-TK e.K.) to allow the air to circulate. After 24 h the adult beetles were separated from the flour using a 710 μm mesh size sieve (Retsch). The flour was subsequently separated from the eggs using a 280 μm mesh size sieve.

The following egg bleaching and washing procedures were performed in a sterile environment. Four micro-spoons (Sigma-Aldrich) of eggs were placed in a cell strainer with a 40 μm nylon mesh (BD Biosciences). The cell strainer containing the eggs was soaked and rinsed in 6% NaClO solution for 3 min and subsequently washed with sterilized, deionized water two times for 3 min. The cell strainer containing the bleached and washed eggs was placed upside down on an open, sterilized glass Petri dish (150 × 15 mm) and left to dry in a sterile environment for 5 min. The total number of cell strainers with bleached eggs in both experiments was nine and 24, respectively. In Experiment 1 the nine cell strainers were divided into three glass Petri dishes each containing three cell strainers with the eggs, while in Experiment 2 each of the 24 cell strainers was placed into a separate Petri dish. After drying, the Petri dishes containing the cell strainers and sterilized eggs were closed and sealed with Parafilm M^®^ and kept at standard breeding conditions for 4 days.

### Untreated, Sterilized, and Recolonized Flour Treatments

By the fifth day post oviposition (DPO) the eggs had hatched. The Petri dishes were opened in a sterile environment, the larvae were flicked from the cell strainer into the Petri dish, and placed onto one of three different flour treatment: “untreated,” “sterilized,” or “recolonized.” Each of the three flour treatments was prepared from a single mixture of standard flour. The “untreated” flour was not treated in any way, representing our control treatment. Both the “sterilized” and the “recolonized” flour were gamma irradiated with a single dose of 29 kGy using a ^60^Co radiation source (BBF Sterilisation service GmbH, Kernen-Rommelshausen, Germany) to kill any microbes. The “sterilized” flour was not treated in any way after irradiation. In order to reintroduce the insect-associated microbiota to the gamma sterilized flour, the “recolonized” flour was prepared using the following procedure: 100 larvae and 100 adult beetles randomly chosen from the stock population were left *ad libitum* per 100 g of irradiated flour for 7 days under standard breeding conditions. On the seventh day of the flour conditioning, the animals were sieved from the “recolonized” flour using a sterile 280 μm mesh size sieve.

In Experiment 1 three Petri dishes of larvae were placed into 1.7 L glass jars each containing 300 g “untreated,” “sterilized,” or “recolonized” flour, respectively. In Experiment 2 the larvae from the each of the 24 Petri dishes were placed into one of 24 0.7 L glass jars, eight replicates of either 100 g of “untreated,” “sterilized,” or “recolonized” flour, respectively. The glass jars containing the flour and the newly hatched larvae were sealed with sterile breathable sealing foil for culture plates (Kisker Biotech) and kept under standard breeding conditions for 9 days. On DPO 14 the larvae were separated from the flour using a 280 μm mesh size sieve. After the flour was removed, the remaining larvae were allowed to crawl through a system of sterile sieves with four descending mesh sizes (710, 560, 500, and 280 μm) put on top of one another for 5 min. Uniform-sized larvae that remained in the 500 μm mesh size sieve after 5 min were used for priming.

### Immune Priming

We produced a priming diet and two additional control diets for Experiment 1. They all contained gamma irradiated standard flour plus either supernatant from a *Btt* culture (“primed”), BT medium (“medium control”) or phosphate buffered saline—PBS (“PBS control”). The plates for oral priming were designed in a way that each plate contained all treatments. For Experiment 2 we omitted the PBS control group. All bacterial cultures were grown at 30°C in darkness. The oral priming diet was prepared following a previously described protocol (Milutinović et al., 2014) with a few minor modifications. Briefly, *Btt* from the frozen stock was plated on a LB agar plate and incubated overnight. The following day, 5 mL of BT medium [w/V–0.75% Bacto Peptone (Sigma), 0.1% glucose, 0.34% KH_2_PO_4_, 0.435% K_2_HPO_4_] was supplemented with 25 μL of sterile salt solution (0.2 M MgSO_4_, 2 mM MnSO_4_, 17 mM ZnSO_4_, 26 mM FeSO_4_), 6.25 μL of sterile 1 M CaCl_2_ × 2H_2_O solution, inoculated with three *Btt* colony forming units from the LB agar plate and incubated overnight in culture tubes (Simport) at 200 rpm. The following morning, 300 mL of BT medium were supplemented with 1.5 mL of salt solution, 375 μL 1M CaCl_2_ × 2H_2_O and inoculated with 1 mL of the overnight culture. The culture was incubated in a 2 L Erlenmeyer flask for 6 days at 200 rpm. On the third day of incubation, an additional 1.5 mL of salt solution and 375 μL 1M CaCl_2_ × 2H_2_O were added to the culture. On day six, the culture was centrifuged at 3700 × *g* for 10 min at room temperature (RT). After centrifugation, the supernatant was retained and transferred to a new 50 mL centrifugation tube (Sigma) and the centrifugation step was repeated. After the second centrifugation, the supernatant was transferred to a new 50 mL tube and filter-sterilized using first a 0.45 μm and subsequently a 0.2 μm cellulose acetate filter (Whatman GmbH). For the primed diet, the sterile supernatant was mixed with 0.15 g of gamma irradiated standard flour per milliliter of supernatant. For the medium control diet, we mixed 0.15 g of gamma irradiated standard flour per milliliter with unconditioned medium (i.e., BT medium plus salts only). For the PBS control diet, designed to control for the effects of the BT medium itself on the larval survival, we used 0.15 g of gamma irradiated standard flour per milliliter of PBS. For both experiments 30 μL of diet (Experiment 1: priming, medium control, PBS control; Experiment 2: priming and medium control) were pipetted into each well of a 96-well plate, and this was replicated 15 times in Experiment 1 and 24 times in Experiment 2. After pipetting the diets, the plates were covered with a sterile breathable sealing foil for culture plates and the flour was allowed to dry for 24 h at 36°C.

The larvae were placed individually into the 96-well plates. In Experiment 1, 360 larvae were placed onto each of the three priming diets. In Experiment 2, 1152 larvae were primed and 1152 were used as a medium control. The plates were sealed with a transparent adhesive tape (Tesa SE) and nine holes were punctured with a needle (0.3 mm in diameter) above each well. The plates were placed in 2.6 L boxes with air circulation vents as described above. Each box contained three 96-well plates. After 24 h on the priming diet (DPO 15) all larvae were individually transferred to new 96-well plates containing only PBS diet. Note that this PBS diet step was applied to all larvae for 4 days, regardless of the previous priming treatment they received. The plates were sealed and put into new boxes with air circulation vents as described above and kept at standard breeding conditions for 4 days until challenge, i.e., DPO 19.

### Immune Challenge

We produced two diets: the “challenge” diet contained *Btt* spores and the “control” diet contained no spores. The diet for the oral challenge was prepared following the same protocol as for the priming and as previously described (Milutinović et al., 2013) with a few minor modifications. The volume of the *Btt* spore cultures was 600 mL and they were incubated in a 2 L Erlenmeyer flask. On the sixth day of spore culturing, spores were harvested by centrifugation at 3700 × *g* for 10 min on RT. The supernatant was discarded, the spore pellets from all the centrifugation tubes were resuspended with 2 mL of PBS and pooled into a single 50 mL centrifugation tube. The spores were centrifuged (3700 × *g* for 10 min at RT), the supernatant was discarded, and the spore pellet was resuspended in PBS. The spore concentration of the diet was adjusted to 1 ×10^10^ mL^-1^ by adding PBS, and 0.15 g of gamma irradiated standard flour was added per milliliter of spore suspension. In both experiments 30 μL of spore containing challenge diet and 30 μL of medium control diet were pipetted into each half of the 96-well plate. Such a plate was replicated 15 times in Experiment 1 and 24 times in Experiment 2. The plates were covered, placed in 2.6 L plastic box with air circulation vents as described above and dried for 12 h at 50°C.

On DPO 19, larvae were individually transferred onto the challenge diet. The total number of larvae challenged per each of the 18 flour/priming/challenge treatments for Experiment 1 ranged between 101 and 108 (a few larvae died and/or escaped during priming), for Experiment 2, 96 larvae were used for each of the 12 flour/priming/challenge treatments. The larvae were kept on the challenge diet for 7 days and the survival was checked every 24 h.

### Assessing Larval Bacterial Load After Exposure to Flour Treatments

To check the larval bacterial load, on DPO 14, ten similar-size larvae were pooled into a 1.5 mL tube (Eppendorf) from each jar in three replicates per jar and 500 μL of RNA later RNA Stabilization Reagent (Qiagen) were added. The larvae that had been removed from the experiment on DPO 14 were tested via real time quantitative PCR (RT-qPCR) for bacterial cDNA as an indicator of the level of bacteria in the larval guts after exposure for 9 days to one of the three flour treatments. The RNA later reagent was removed from the tubes and the larvae were homogenized under sterile conditions, on ice, using a sterile pestle. The total RNA from the *T. castaneum* larvae and associated bacteria was extracted using a PowerMicrobiome^TM^ RNA Isolation Kit (MO BIO Laboratories, Inc.) following the manufacturer’s protocol. The concentration of the extracted RNA was measured using a Qubit^®^ 2.0 Fluorometer and the corresponding Qubit RNA HS Assay Kit (Life Technologies GmbH) following the manufacturer’s instructions. The concentration of the extracted RNA was equalized across samples in both experiments. The cDNA was synthesized using the SuperScript^®^ III First-Strand Synthesis System (Life Technologies GmbH) and random hexamer primers (Life Technologies GmbH) following the manufacturer’s protocol. The relative quantification of bacterial cDNA from the 16S rRNA gene was performed using a real-time quantitative PCR system LightCycler^®^ 480 (Roche) with the KAPA^TM^ SYBR^®^ Green I chemistry. The primers used to amplify the hypervariable region V5-V6 of the bacterial 16S rRNA cDNA were fwd: 799F-mod2 (5′ AACMGGATTAGATACCCKGGT 3′) and rev: 1114R (5′ GCAACGAGCGCAACCC 3′) (Hanshew et al., 2013) yielding a PCR product of approximately 315 bp. The size of the amplicons was confirmed on an agarose gel (data not shown). Relative quantification results were normalized with a *T. castaneum rp49* gene as an internal standard. The primers used for amplification of the *rp49* gene were fwd: (5′ TTATGGCAAACTCAAACGCAAC 3′) and rev: (5′ GGTAGCATGTGCTTCGTTTTG 3′) (Eggert et al., 2014). All the qPCR measurements were made in duplicates. The qPCR reactions were performed in 20 μ L reaction volumes and the following protocol was used: pre-incubation 95°C for 3 min followed by 40 cycles of 10 s at 95°C, 20 s at 58°C and 2 s at 72°C. The fluorescence acquisition was performed in each cycle at 72°C. In order to confirm the identity of the PCR products, a melting curve was derived using the temperature range between 95 and 58°C.

### Survival Analyses

Larval survival over the 7 days following challenge was analyzed using the R statistical package for Macintosh (RStudio version 0.99.441) (R Core Team, 2013). Within R we used Cox proportional hazards models and mixed-effects Cox proportional hazards models with the following packages: “bdsmatrix” (Therneau, 2013a), “coxme” (Therneau, 2012), “matrix” (Bates and Maechler, 2013), “nlme” (Pinheiro et al., 2013), “splines” (R Core Team, 2013), and “survival” (Therneau and Grambsch, 2000; Therneau, 2013b); the latter allows for the inclusion of random effects where necessary. All models were fitted with day of death as the response variable: larvae that were alive at the end of the experiment were included as censored cases.

For Experiment 1 we analyzed each of the flour treatments (“untreated,” “sterilized,” and “recolonized”) separately. For each of these three flour treatments we performed three separate models, which were designed to test: 1. whether there was a significant overall effect of the factor “challenge” (two levels: control and challenge); 2. within the control group (i.e., those that did not receive *Btt* spores at challenge), whether there was a significant effect of the factor “priming” (three levels: medium control, PBS control, primed); 3. within the challenge group (i.e., those that did receive *Btt* spores at challenge) whether there was a significant effect of priming. This gave us nine models in total for Experiment 1.

For Experiment 2 we took a similar approach, except that priming only had two levels (primed and medium control) and we included jar as a random effect. The “coxme” models including jar were compared to “coxph” models (without this random effect) in order to determine whether there was a significant effect of jar. Jar was removed during model reduction as in all cases it was non-significant.

In both experiments, in all cases where the Cox proportional hazards models did not fulfill the assumptions of proportional hazards over time (based on Schoenfeld residuals) we also tested the model using the survival regression (“survreg”) function in R. In all cases the results from the Cox proportional hazards and survival regression were qualitatively similar, therefore we present the results of the Cox proportional hazards models.

### RT-qPCR Data Analysis

The results of the relative quantification of bacterial 16S rRNA across different jars were analyzed using the Relative Expression Software Tool, REST 2009 (Pfaﬄ et al., 2002). The *T. castaneum rp49* gene was used as the internal standard. Levels of 16S rRNA cDNA of “sterilized” and “recolonized” larvae were compared to the “untreated” larvae. Also, “sterilized” larvae were compared to “recolonized” larvae. Primer efficiency used in the analysis was 90% for the target gene and 95% for the internal standard.

### Ethical Statement

According to the German Animal Welfare Act, experimental work on insects is not a subject to any ethical approval. Nevertheless, all the experiments in this study were performed in accordance with good ethical and scientific practice.

## Results

### Microbial Load of Larvae

**FIGURE 1:**
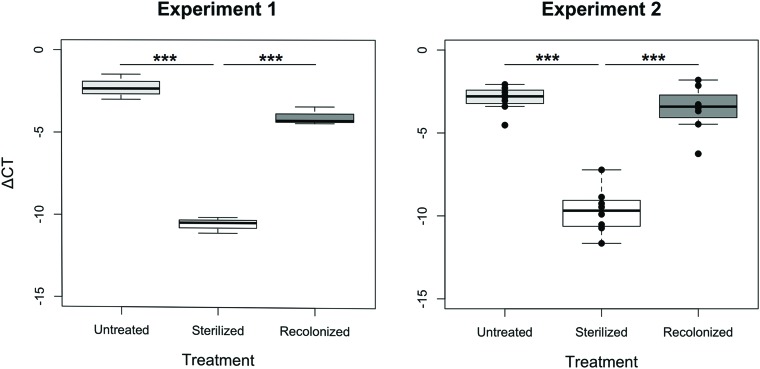
**Bacterial load of larvae raised on “untreated,” “sterilized,” and “recolonized” flour treatments, expressed as a ΔCT value of the bacterial *16S rRNA* gene relative to the *T. castaneum* reference gene, *rp49*.** Experiment 1 is based on three samples (each containing ten pooled larvae) per flour treatment, while Experiment 2 is based on eight independently produced flour replicates per treatment. From each of those, three samples (of ten pooled larvae each) were analyzed and the respective mean ΔCT values are indicated by the dots. Boxplots show the overall median and quartiles, whiskers indicate standard errors. Statistically significant differences are indicated by the asterisks (*p* < 0.001).

In both experiments, the sterilization and recolonization treatments of the flour were successful: larvae reared on the “sterilized” flour treatment, although not entirely free of bacteria or their nucleic acids that may be amplified in the qPCR, showed a significantly lower qPCR signal compared to larvae reared on both “untreated” and “recolonized” flour (*p <* 0.001, **Figure 1**). Although the larvae raised on the “recolonized” flour also showed somewhat lower bacterial load estimates, these levels were not significantly lower than the “untreated” larvae (Experiment 1, *p* = 0.068; Experiment 2, *p* = 0.101; **Figure 1**).

### Survival After Priming and Challenge Depends Upon the Larval Flour Treatment

**FIGURE 2:**
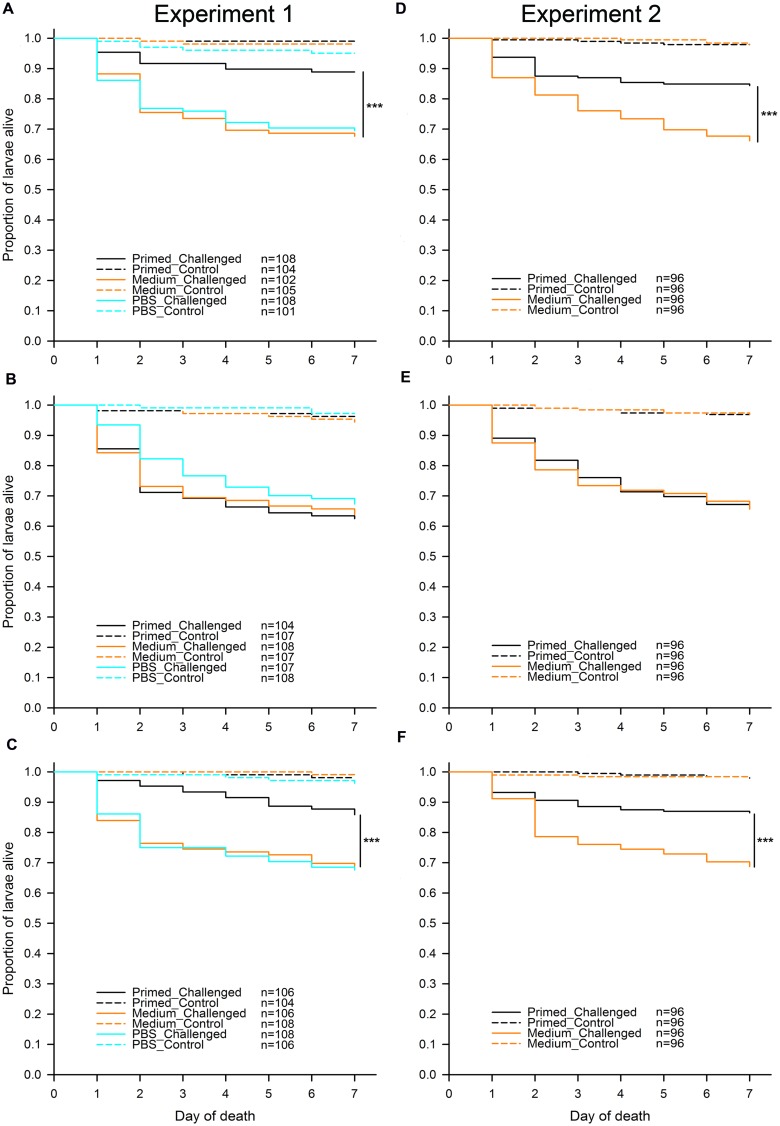
**Effect of microbiota on *T. castaneum* survival after oral immune priming and challenge for Experiments 1 and 2.** Survival of larvae raised on the following flour treatments: “untreated”—**(A,D)**, “sterilized”—**(B,E)**, “recolonized”—**(C,F)**. Priming treatments were primed with *Btt* spore culture supernatants (“Primed”), unconditioned spore culture medium (“Medium”) or phosphate-buffered saline (“PBS”) and subsequently challenged with *Btt* spores (“Challenged”) or PBS (“Control”). Sample sizes for each experimental group are shown in the treatment legends. Panels **(D–F)** (Experiment 2) show the cumulative data from 8 jar-replicates per flour treatment. Statistically significant differences are indicated by the asterisks (*p ≤* 0.001).

To address the question of whether different flour treatments (“untreated,” “sterilized,” and “recolonized”) led to differences in priming, we analyzed each of the flour treatments in both experiments separately (**Figure 2**). In all flour treatments, as predicted, larvae challenged with *Btt* spores showed a significantly lower survival than control larvae, which were not exposed to any spores. For Experiment 1 the results were: “untreated” (*χ*^2^ = 73.33, df = 1, *p <* 0.001), “sterilized” (*χ*^2^ = 113.43, df = 1, *p <* 0.001), “recolonized” (*χ*^2^ = 86.39, df = 1, *p <* 0.001), while the results of Experiment 2 reflected the results from the first experiment: “untreated” (*χ*^2^ = 98.88, df = 1, *p <* 0.001), “sterilized” (*χ*^2^ = 139.53, df = 1, *p <* 0.001), “recolonized” (*χ*^2^ = 88.85, df = 1, *p <* 0.001). This confirmed that the larvae exposed to *Btt* spores indeed died from *Btt* infection.

Since the results of the individual comparisons from Experiment 1 had shown no difference in survival between the medium and PBS control larvae regardless of the flour treatment: “untreated” (*z* = -0.236, *p* = 0.813) and “recolonized” (*z* = 0.161, *p* = 0.872), only the medium control was included into Experiment 2, while the PBS control was omitted. Potential differences among the eight replicate jars for each flour treatment, which were used in Experiment 2 in order to increase the statistical power of the experiment, were initially included as a random effect into the statistical analyses, but were removed during model reduction, indicating consistency among the replicates.

As expected, for both experiments and across all treatments without *Btt* spores, on average very few (2.49%) control larvae died. Among control larvae in both experiments, there was no effect of the priming treatment on survival: Experiment 1, “untreated” (*χ*^2^ = 3.40, df = 2, *p* = 0.182), “sterilized” (*χ*^2^ = 1.12, df = 2, *p* = 0.572), “recolonized” (*χ*^2^ = 2.09, df = 2, *p* = 0.352), and Experiment 2, “untreated” (*χ*^2^ = 0.00, df = 1, *p* = 0.990), “sterilized” (*χ*^2^ = 0.00, df = 1, *p* = 0.994), “recolonized” (*χ*^2^ = 0.13, df = 1, *p* = 0.711). Control larvae were thus excluded from the further analyses.

In contrast to the control larvae, in Experiment 1 there was a significant overall effect of priming on survival of the challenged larvae when reared on “untreated” flour (*χ*^2^ = 17.49, df = 2, *p <* 0.001). Pairwise comparisons showed that, as predicted, challenged larvae previously primed with *Btt* supernatant had significantly higher survival than the medium control primed larvae (*z* = -3.527, *p <* 0.001), i.e., the larvae showed the expected priming effect (**Figure 2A**). The same expected priming effect was observed in larvae raised on the “untreated” flour in Experiment 2: (*χ*^2^ = 16.49, df = 1, *p <* 0.001; **Figure 2D**). Sterilizing the flour removed the effect of priming both in Experiment 1 (*χ*^2^ = 1.03, df = 2, *p* = 0.598; **Figure 2B**) and Experiment 2 (*χ*^2^ = 0.03, df = 1, *p* = 0.863; **Figure 2E**). Recolonizing the sterilized flour with microbes resulted in the re-emergence of a significant priming effect in Experiment 1 (*χ*^2^ = 13.50, df = 2, *p* = 0.001; **Figure 2C**). Similarly to the “untreated” flour, challenged larvae previously primed with *Btt* supernatant had significantly higher survival than the medium control primed larvae (*z* = -3.003, *p* = 0.003). The same expected priming effect was reestablished when larvae were raised on the “recolonized” flour in Experiment 2 (*χ*^2^ = 16.82, df = 1, *p <* 0.001; **Figure 2F**).

## Discussion

Our results show that host-associated microbiota plays a crucial role in the mediation of oral priming in *T. castaneum* with *Btt:* priming larvae with *Btt*-derived components only provided protection against a subsequent exposure to *Btt* spores when the microbiota was present in the larval gut. Our treatment where larvae whose microbiota had previously been removed, but which could subsequently obtain environmental microbes by feeding on untreated flour, showed that priming occurs via oral exposure, thereby confirming previous results on oral immune priming in the same beetle population and bacterial strain showing that priming occurs via oral exposure (Milutinović et al., 2014). By contrast, we found no priming when larvae where not allowed to regain any microbes, i.e., reared on a diet of gamma-sterilized flour. It is noteworthy that a low level qPCR signal was observed even in larvae from the “sterilized” treatment (**Figure 1**), which could indicate the presence of some radiation resistant bacteria (Cox and Battista, 2005) or amplified DNA residues from killed bacteria (Trampuz et al., 2006). Importantly, we were able to reestablish the priming effect when such larvae were instead reared on gamma-sterilized flour that had been subsequently recolonized with microbes from conspecifics. That priming was possible when the larvae obtained their gut microbes in different manners (i.e., “untreated” and “recolonized” flour treatments) illustrates the robustness of oral immune priming that was previously demonstrated without any manipulation of microbes (Milutinović et al., 2014).

Our results are in accordance with the *A. gambiae – P. falciparum* host-parasite system, where Rodrigues et al. (2010) found oral immune priming only in the presence of commensal gut-harbored bacteria. *Plasmodium* ookinetes invade the mosquito midgut by disruption of the peritrophic membrane, which is otherwise impermeable to gut microbes. Using the damage caused by ookinetes, gut bacteria can invade the midgut epithelial cells triggering an immune response, which leads to a long-lasting antibacterial priming based on quantitative and qualitative hemocyte differentiation (Rodrigues et al., 2010). However, when the gut microbiota was removed from the mosquito, the priming effect disappeared. A lipoxin/lipocalin complex was recently identified as a mediator of such priming in the mosquito immune system. *Plasmodium* ookinete midgut invasion induces the release of the lipoxin/lipocalin complex that acts as a hemocyte differentiation factor (Ramirez et al., 2015). Despite clear differences in biology and ecology between a dipteran being parasitized by a protozoan and a bacterial parasite in a coleopteran host used in our study, we find it intriguing that microbiotas are associated to immune priming in both examples.

While the insect immune enzyme phenoloxidase seems to play a role in paternal trans-generational immune priming upon cuticle pricking in our model organism (Roth et al., 2010; Eggert et al., 2014), the precise mechanism involved in oral priming of the *T. castaneum* immune system is currently unknown. Rather than a single, universal mechanism for priming, it appears more likely that priming might rely on different mediators that could be contingent upon the route of infection (Behrens et al., 2014) and the peculiarities of the interplay between the specific type of pathogen, the gut microbes and the host immune system.

In our host–pathogen system, we can envision a number of possibilities as to how oral priming could depend on the microbiota. First, reminiscent of the mosquito-malaria example (Rodrigues et al., 2010), priming might result from gut microbiota invading the hosts’ epithelium or body cavity, for example with the help of *Bt* Cry toxins, the most important virulence factor of *Bt*, or some other factors. Encoded on plasmids, *Bt* Cry toxins are expressed during sporulation and form large crystalline inclusions, which show specific entomotoxicity. Binding specifically to receptors of the midgut epithelium of the host, Cry toxins cause cell lysis and extensive damage to the host (Soberón et al., 2009). Although no bacterial cells or spores, nor Cry toxin crystals were observed in the bacteria-conditioned medium after filter-sterilization that we used for oral priming (incubation and microscopic evaluation, data not shown), potential Cry toxin monomers or crystals smaller than the pores of the filter used for filter-sterilization of the *Btt* culture supernatant (0.2 μm) might still be present. It is noteworthy in this context that a role of the midgut bacteria for susceptibility to *B. thuringiensis* was controversially discussed for lepidopteran species (Broderick et al., 2006, 2009, 2010; Raymond et al., 2010). However, it should also be noted that in contrast to priming, infection itself did not depend on microbiota in our system, i.e., killing of *T. castaneum* by *Btt* (after non-effective priming with medium or PBS) was similar across flour treatments, with or without microbiota (**Figure 2**, orange and turquoise solid lines).

Second, priming might directly change the abundance or composition of the gut microbiota, which could then lead to enhanced resistance. There are numerous examples for a role of the microbiota in insect resistance (reviewed in Engel and Moran, 2013; Garcia-Garcia et al., 2013). For example, the microbiome of *Glossina* spp. modulates the host’s ability to mount an effective immune response after a challenge with trypanosome parasites (Weiss et al., 2013). Furthermore, selecting for increased lytic activity and resistance to a *B. thuringiensis* based product (Xentari) in the lepidopteran species *Spodoptera exigua* resulted in host lines with increased midgut microbial loads and higher antimicrobial immunity (Hernández-Martínez et al., 2010). Apart from a general protection against diverse infections in *B. terrestris* workers (Koch and Schmid-Hempel, 2011), the composition of gut microbiota is responsible for specific resistance against different pathogen strains (Koch and Schmid-Hempel, 2012). Antagonistic interactions between bacterial species in the gut could for example lead to reduced spore germination or vegetative growth of *Btt.* In this context, it is relevant that we recently found reduced *Btt* spore germination for *T. castaneum*-derived, as compared to laboratory-raised, *Btt* spores (Milutinović et al., 2015), although we did not test whether this effect is dependent on host microbiota. More generally, it should be noted that an effect of gut organisms and their interactions on priming might not be restricted to bacteria, but could also involve archaea, fungi or viruses, or even eukaryotic parasites, such as gregarines, whose negative effect on trans-generational immune priming was recently demonstrated in *Tribolium confusum* (Tate and Graham, 2015). However, such interactions would need to be tested in future studies.

Third, priming-induced immune responses might lead to differences in the gut microbiota that could then mediate resistance upon challenge. It is known that the tightly regulated expression of antimicrobial peptides (AMP) in the gut of *D. melanogaster* plays an essential role for the maintenance of the gut microbiota, which in turn is crucial for host health (Ryu et al., 2008). Once the expression of AMP genes was experimentally increased, the microbial equilibrium in the gut was disturbed resulting in an abnormal dominance of a single strain of *Gluconobacter* sp. which led to an apoptosis of the gut epithelial cells and death of the host (Ryu et al., 2008).

Finally, it is possible that the removal of the indigenous gut microbiota is more generally detrimental for the host, especially if symbionts are needed for certain metabolic functions (reviewed, in Engel and Moran, 2013). A reduced general body condition might lead to a lack of immunocompetence necessary for building up a primed immune response. However, little is currently known about the composition and role of the *T. castaneum* microbiota (Kumari et al., 2011).

Whatever the mechanism, our study contributes to the still limited evidence for a role of microbes in immune priming. Importantly, this does not imply that microbes are always necessary for the host to achieve a primed state of the immune system (see e.g., Contreras-Garduño et al., 2015). We rather think that there could be a multitude of different mechanisms underlying priming in the different host–pathogen systems, and that only a few of those might be microbe-dependent. Further studies are needed to shed light on the role that microbes play in this fascinating form of memory in invertebrate immunity.

## Author Contributions

MF, SA, and JK conceived and designed the experiments. MF performed the experiments. MF, SA, and JK analyzed the data, wrote and revised the manuscript and approved the final version for publishing.

## Conflict of Interest Statement

The authors declare that the research was conducted in the absence of any commercial or financial relationships that could be construed as a potential conflict of interest.
